# Implementation strategies for integrating non-communicable disease care into primary healthcare settings in sub-Saharan Africa: A systematic review protocol

**DOI:** 10.1371/journal.pone.0356003

**Published:** 2026-08-14

**Authors:** Hubert Amu, Theodora Yayra Brinsley, Joshua Oppong

**Affiliations:** 1 Department of Population and Behavioural Sciences, Fred Newton Binka School of Public Health, University of Health and Allied Sciences, Hohoe, Ghana; 2 Department of Epidemiology and Disease Control, School of Public Health, University of Ghana, Accra, Ghana; Indiana University South Bend, UNITED STATES OF AMERICA

## Abstract

**Background:**

Non-communicable diseases (NCDs) account for a large and growing share of global mortality, with the highest burden in low- and middle-income countries (LMICs). The World Health Organization and global partners emphasise strengthening primary health care (PHC) as a central pathway toward universal health coverage (UHC). The WHO Package of Essential Noncommunicable Disease Interventions (PEN) for PHC explicitly positions “integration of NCD management into primary health care” as an important step, particularly in LMICs. Despite the wide policy endorsement, evidence suggests there are ongoing gaps in integrating essential NCD care into PHC across Africa.

**Methods:**

This protocol follows PRISMA-P guidance for systematic review protocols. The review will include peer-reviewed and grey literature (English) from 1 January 1990 to 31 December 2025 that focuses on implementation strategies for integrating NCD care into PHC in sub-Saharan Africa (SSA). Narrative synthesis will be conducted using established guidance for narrative synthesis and SWiM reporting, where meta-analysis is not feasible. Study quality will be appraised using the Mixed Methods Appraisal Tool (MMAT) to accommodate heterogeneous designs.

**Ethics and dissemination:**

Ethical approval is not required because the review will use publicly available data only. Findings will be disseminated through peer-reviewed publications and conference presentations.

**Registration:**

This protocol was registered with PROSPERO (CRD420261354803). https://www.crd.york.ac.uk/PROSPERO/view/CRD420261354803.

**Protocol reporting standard:**

This protocol is prepared in accordance with PRISMA-P (Preferred Reporting Items for Systematic Reviews and Meta-Analyses Protocols) 2015, which provides a minimum set of items for transparent reporting of systematic review protocols.

**Amendments:**

Any protocol amendments (e.g., eligibility criteria refinements or database additions) will be documented with date, rationale, and impact on methods, and updated in the PROSPERO record and the final manuscript.

## Introduction

Non-communicable diseases (NCDs) such as cardiovascular diseases, diabetes, cancers, chronic respiratory conditions, and mental health disorders are the leading causes of mortality. Globally, NCDs account for three-quarters of all deaths [1]. Low- and middle-income countries (LMICs), especially in SSA, bear this burden due to rapid urbanisation, demographic shifts, and lifestyle changes, which significantly contribute to the rise in chronic disease prevalence [2,3]. SSA is consequently experiencing a complex epidemiological transition characterised by a dual burden of infectious diseases and NCDs, placing significant strain on already resource-constrained health systems.

Historically, health systems in SSA have been organised around vertical, disease-specific programmes, which are supported by external funding targeting communicable diseases such as HIV/AIDS, tuberculosis, and malaria [4]. These programmes have contributed to strengthening public health but have also resulted in fragmented service delivery structures that are not well-suited to the long-term and coordinated care required for NCD management. The fragmentation makes it difficult to manage NCDs. Many patients with chronic diseases must visit different clinics or programmes to obtain tests, medicines, and follow-up care, leading to gaps in treatment [5,6]. Strengthening PHC can resolve these challenges through its emphasis on comprehensive, continuous, and person-centred care. Integrating NCD services into PHC would allow patients to receive more coordinated and continuous care through a single point of service, reduce duplication, and ensure that patients with multiple chronic conditions receive coordinated management. Individuals living with chronic conditions face challenges, including limited access to essential diagnostics and medications, poor continuity of care, weak referral systems, and inadequate follow-up, particularly at the PHC level [7].

These health-system challenges are reflected in the health-seeking behaviour of individuals living with NCDs, and the available evidence reinforces the extent of this gap at the PHC level. In rural Ghana, patients with hypertension and diabetes have been found to favour public hospitals over community health centres [8], while fewer than a quarter of rural Burkina Faso’s chronic-disease patients access any formal facility-based care [9]. This underutilisation is associated with barriers including cost, distance, and broader health-system constraints [10]. This pattern of underusing PHC facilities suggests that PHC has yet to fulfil its intended role as the first point of contact for NCD prevention and management.

Primary healthcare is the most equitable and sustainable platform for delivering comprehensive health services. The 2018 Declaration of Astana reaffirmed global commitment to PHC as the foundation for achieving Universal Health Coverage (UHC) [11]. This emphasises the need for integrated, people-centred, and continuous care across the life course and also reinforces the relevance of the World Health Organization (WHO) Package of Essential Noncommunicable Disease Interventions (WHO PEN), which promotes the delivery of cost-effective NCD services at the primary care level [12]. These global frameworks reflect the need to identify effective implementation strategies for integrating NCD care into PHC systems to improve equitable, accessible, and comprehensive care.

Progress in integrating NCD care into PHC across SSA has been uneven despite the strong policy endorsements. Variations in health system capacity, workforce availability, infrastructure, governance, and financing mechanisms continue to influence the extent and effectiveness of integration efforts [13]. A range of implementation strategies has been employed to facilitate this integration, including task-shifting, decentralisation of services to enhance access, adoption of integrated chronic care models, utilisation of digital health technologies such as mobile health (mHealth) and telemedicine, and strengthening of referral and supply chain systems [14,15]. However, these strategies are highly context-dependent, and their outcomes vary across different geographic locations.

The existing evidence base on implementation strategies for integrating NCD care into PHC in SSA is fragmented and heterogeneous. Studies differ in design, scope, intervention types, and outcome measures [16,17]. This encompasses quantitative evaluations of service delivery and clinical outcomes, qualitative explorations of implementation processes and contextual factors, and mixed-methods approaches that attempt to bridge these perspectives. This diversity presents challenges for synthesis and limits the ability of policymakers and practitioners to identify effective, scalable, and sustainable models of integration.

The integration of NCD care into PHC systems aligns with global development priorities, specifically Sustainable Development Goal (SDG) 3.4, which aims to reduce premature mortality from NCDs through prevention and treatment, and SDG 3.8, which focuses on achieving universal health coverage, including access to quality essential healthcare services and affordable medicines [18]. Strengthening PHC systems to deliver integrated NCD services also supports broader development objectives, including reducing health-related financial hardship (SDG 1), addressing inequalities in access to care (SDG 10), and promoting partnerships for health system strengthening (SDG 17) [18].

Given the rising burden of NCDs and the central role of PHC in addressing this burden, there is a need to synthesise evidence on how integration is being implemented in SSA and what strategies are most effective within different contexts. Previous reviews have examined models of care or specific interventions [19,20], but there is a gap in systematically analysing implementation strategies, including their characteristics, contextual determinants, and outcomes.

This systematic review, therefore, seeks to address the following research questions: What implementation strategies have been used to integrate NCD care into primary healthcare in SSA, and what are their associated barriers, facilitators, and effects on service delivery and patient outcomes? To answer this question, the review aims to identify the implementation strategies used to integrate NCD care into PHC settings in SSA, examine the barriers and facilitators influencing their implementation, assess the reported effects of these strategies on service delivery and patient outcomes, and analyse their implications for scale-up, sustainability, and health system strengthening within primary healthcare systems in the region.

Synthesising evidence across diverse study designs and contexts, this review adopts an implementation-focused perspective that goes beyond assessing effectiveness to understanding how and why integration strategies succeed or fail. The findings could provide evidence for policymakers and health system stakeholders, thereby contributing to efforts aimed at strengthening PHC systems and advancing progress toward universal health coverage and improved NCD outcomes in SSA.

## Materials and methods

### Design and methodological standards

Our study will be conducted as a systematic review of qualitative, quantitative, and mixed-methods evidence examining implementation strategies for integrating NCD care into PHC in SSA. The review will be reported in accordance with the Preferred Reporting Items for Systematic Reviews and Meta-Analyses (PRISMA) 2020 [21] guidelines for the final manuscript, while this protocol has been developed following the Preferred Reporting Items for Systematic Reviews and Meta-Analyses Protocols (PRISMA-P) 2015 statement to ensure transparency and reproducibility [22] S1 Table.

Given the anticipated heterogeneity in study designs, intervention types, and outcome measures, synthesis and reporting will align with the Synthesis Without Meta-analysis (SWiM) guidance to ensure clarity and transparency in narrative reporting [23]. The conduct of the review, including literature searching, study selection, data extraction, and synthesis, will be guided by methodological principles outlined in the Joanna Briggs Institute (JBI) Manual for Evidence Synthesis [24]. Any amendments to the registered protocol during the course of the review will be documented, justified, and reported transparently in both the PROSPERO record and the final publication.

### Study dates

The review commenced on 15 March 2026, with protocol registration and database searches. Preliminary database searches have been initiated; however, study screening, data extraction, and analysis have not yet commenced. We anticipate completing study screening, data extraction, and analysis by 28 May 2026, with final manuscript submission expected by June 2026.

### Eligibility criteria

#### Geographic scope.

The review will include studies conducted in SSA, as defined by the World Bank SSA region. Multi-country studies will be included if at least one participating country falls within this classification and results are reported for SSA contexts.

### Time window and language

We will review studies published in English between 1 January 1990 and 31 December 2025. This timeframe has been selected to capture both early and contemporary efforts toward integrating NCD care into PHC systems.

### PEO framework

#### Population.

Our population will include studies conducted among individuals receiving NCD-related services, including prevention, screening, diagnosis, treatment, follow-up, and chronic care within PHC settings. It will also include health workers or PHC teams involved in delivering NCD care, such as medical doctors, nurses, midwives, community health workers, physician assistants, and other non-physician providers.

### Exposure

Our exposure of interest will be implementation strategies or delivery models aimed at integrating NCD care into PHC. These strategies may include task-shifting and task-sharing approaches, decentralisation of services, integrated clinic models, implementation of clinical guidelines and protocols, workforce strengthening interventions, strengthening of referral pathways, digital health and decision-support tools such as mHealth and telemedicine, and community-based PHC programmes targeting NCDs.

Where data permit, implementation strategies will be described using recommended reporting dimensions, including the actor, action, target, temporality, and dose, to improve interpretability and comparability. The Expert Recommendations for Implementing Change (ERIC) taxonomy may be used as a reference to standardise the classification of strategies where feasible [25].

### Outcomes

Studies will be eligible if they report on the implementation, application, or evaluation of at least one strategy for integrating NCD care into PHC settings in SSA. The primary outcome of interest is the implementation strategy. This includes the description, actors, actions, setting, and duration of application. Secondary outcomes include implementation outcomes (acceptability, adoption, appropriateness, feasibility, fidelity, cost, penetration, and sustainability); service delivery outcomes (access, coverage, continuity of care, quality indicators, adherence to guidelines, referral completion, and supply chain performance); and patient-level clinical outcomes (blood pressure control, glycaemic control, retention in care, treatment adherence, complication rates, and patient experience). Studies focused solely on NCD epidemiology or clinical effectiveness without describing an implementation or integration strategy will be excluded.

### Types of studies

Our review will include a broad range of empirical study designs to capture the complexity of implementation processes. These will include randomised controlled trials and quasi-experimental studies evaluating integration strategies, observational studies (cross-sectional, cohort, and interrupted time series designs), and qualitative studies exploring implementation experiences, barriers, facilitators, acceptability, and feasibility. The synthesis will also include mixed-methods studies evaluating implementation strategies.

Studies will be excluded if they are conducted exclusively in secondary or tertiary care facilities without a PHC integration component. Studies focusing solely on NCD epidemiology or clinical effectiveness without an implementation or integration strategy will also be excluded. Editorials, commentaries, and opinion pieces will be excluded, except where they are used explicitly for contextual background and clearly identified as non-empirical sources.

### Information sources

Studies will be obtained from electronic databases, namely PubMed/MEDLINE, Scopus, Web of Science, African Journals Online (AJOL), and Google Scholar, covering all records from 1 January 1990 to 31 December 2025. To improve the retrieval of Africa-focused evidence and reduce publication bias, supplementary searches will include African Index Medicus, where accessible, as well as targeted searches of organisational and policy repositories such as those of the World Health Organization, ministries of health, and implementing partners. These supplementary searches will be conducted using transparent and reproducible procedures.

### Search strategy development

A comprehensive and systematic search strategy will be developed to identify all relevant studies examining implementation strategies for integrating non-communicable disease (NCD) care into primary healthcare (PHC) settings in sub-Saharan Africa (SSA). The search strategy will combine controlled vocabulary (e.g., Medical Subject Headings [MeSH] in PubMed) with free-text keywords to maximise sensitivity and ensure comprehensive retrieval of relevant literature. To enhance transparency and reproducibility, the search strategy will be reviewed by an experienced health sciences librarian to refine the search strings. This will ensure the inclusion of appropriate keywords, synonyms, Boolean operators, truncations, and indexing terms across databases.

The search strategy will be structured around four core concept blocks derived from the PEO framework: (i) non-communicable diseases, (ii) primary healthcare, (iii) integration and implementation strategies, and (iv) sub-Saharan Africa. Within each concept block, synonymous terms and related keywords will be combined using the Boolean operator **“OR”** to broaden the search, while the four concept blocks will be combined using the Boolean operator **“AND”** to ensure that retrieved studies address all relevant domains.

For the NCD concept, search terms will include both general and condition-specific terminology. These will include “noncommunicable diseases,” “non-communicable diseases,” “chronic disease,” “chronic diseases,” “chronic illness” and the abbreviation “NCD,” as well as specific conditions such as “hypertension,” “high blood pressure,” “diabetes,” “diabetes mellitus,” “cardiovascular disease,” “stroke,” “cancer,” “neoplasm,” “chronic respiratory disease,” “chronic obstructive pulmonary disease,” “COPD,” “asthma,” “mental health,” “mental disorder,” “depression,” and “anxiety disorder.” These terms will be combined using “OR” to ensure comprehensive coverage of all major NCD categories.

For the PHC concept, keywords will include “primary health care,” “primary healthcare,” “primary care,” “community health services,” “community health service,” “community health center,” “community health centre,” “family practice,” “general practice,” and “PHC.” These terms will also be combined using “OR” to capture variations in terminology across different contexts and study designs.

For the integration and implementation concept, the search will adopt terms related to both service integration and implementation processes. Keywords will include “integrated care,” “service integration,” “integrated service delivery,” “care integration,” “integration,” and “continuity of care,” as well as implementation-focused terms such as “implementation,” “implementation strategy,” “implementation strategies,” “implementation research,” “scale up,” “scale-up,” “sustainability,” “task shifting,” “task sharing,” “decentralisation,” “decentralization,” “guideline implementation,” “quality improvement,” “health system strengthening,” “community-based intervention,” “community-based care,” “digital health,” “mHealth,” “mobile health,” and “telemedicine.” These terms will be combined using “OR” to capture the breadth of implementation approaches relevant to PHC integration.

For the geographic concept, the search will include both controlled vocabulary and free-text terms to capture studies conducted in SSA. Keywords will include “sub-Saharan Africa,” “sub Saharan Africa,” “Africa South of the Sahara,” and the names of individual countries within SSA (Ghana, Nigeria, Kenya, Uganda, Tanzania, Ethiopia, Malawi, Mozambique, South Africa, Rwanda, Zambia, Zimbabwe, Cameroon, Senegal, Sierra Leone, Liberia, Namibia, Botswana, Lesotho, and Eswatini). These terms will be combined using “OR” to maximise the retrieval of region-specific studies.

The final search string will combine the four concept blocks using the Boolean operator “AND,” ensuring that all retrieved studies address NCDs, PHC, implementation/integration strategies, and the SSA context. Truncation symbols (e.g., “*”) will be used where appropriate to capture variations in word endings, and phrase searching (using quotation marks) will be employed to improve precision S1 Text.

### Study records and management

All identified records will be imported into the reference management software Zotero for de-duplication. The de-duplicated records will then be uploaded into Covidence to facilitate screening and data extraction processes. All decisions made during the full-text screening stage will be documented, including reasons for exclusion, to ensure transparency and to support the development of a PRISMA flow diagram S1 Fig.

### Selection process

Our study selection will be conducted in two stages. First, TYB and JO will independently screen titles and abstracts of all retrieved records for potential eligibility. Second, the full texts of all potentially eligible studies will be retrieved and independently assessed against the inclusion criteria. Any disagreements at either stage will be resolved through discussion and consensus. Where consensus cannot be reached, HA will be consulted to adjudicate. These screening approaches are intended to minimise selection bias and enhance the reliability of the review process.

### Data extraction

We will use a standardised data extraction form based on JBI [24]. TYB and JO will pilot the form on a subset of included studies before full extraction. The form will be refined iteratively to ensure clarity and completeness. We will extract bibliographic details such as author, year, country, and publication type, and study characteristics (aims, design, sampling, and PHC setting). Information on the NCD conditions addressed will be collected, along with detailed descriptions of the integration model and implementation strategies, including who implemented the strategy, what actions were undertaken, where they were implemented, and over what period.

We will also extract data on barriers and facilitators to implementation, which may be coded inductively or mapped to common categories during synthesis. Outcomes reported in the studies, including implementation, service delivery, and patient outcomes, will also be extracted, along with measurement tools and timepoints.

### Quality appraisal

We will assess the methodological quality of the included studies using the Mixed Methods Appraisal Tool (MMAT) version 2018 [26]. This is suitable for appraising qualitative, quantitative, and mixed-methods studies within a single framework S2 Table. The two reviewers will independently assess each study, and disagreements will be resolved through discussion. Each included study will be evaluated using the five criteria relevant to its design category. We will summarise the quality appraisal findings and present them transparently to contextualise the interpretation of results. No overall numerical score will be calculated unless explicitly required by the publication outlet.

### Data synthesis

Our study will adopt a narrative synthesis approach [27] due to the anticipated heterogeneity in interventions, contexts, and outcome reporting. The synthesis will follow established guidance for narrative synthesis, including developing a preliminary synthesis, exploring relationships within and between studies, and assessing the robustness of findings.

The findings will be organised thematically around types of implementation strategies and integration models, barriers and facilitators across contexts, reported effects on implementation and service delivery outcomes, patient outcomes where available, and implications for scale-up, sustainability, and health system strengthening. Where a subset of studies demonstrates sufficient homogeneity in intervention type, outcome definition, and measurement, a meta-analysis may be conducted using a random-effects model. Statistical heterogeneity will be assessed using the I^2^ statistic. Where pooling is not appropriate, results will be synthesised narratively using clearly defined grouping and comparison strategies.

### Meta-bias and publication bias

Because meta-analysis is not anticipated as the primary synthesis approach, we will assess publication bias qualitatively by examining patterns such as selective outcome reporting, differences between peer-reviewed and grey literature, and gaps in reporting across outcomes and settings. Where meta-analysis is feasible, small-study effects will be explored using appropriate methods such as funnel plot assessment [28].

### Confidence in cumulative evidence

Where quantitative synthesis is conducted, the certainty of evidence will be assessed using the GRADE approach [29]. For qualitative findings, confidence may be assessed using the GRADE-CERQual framework, considering methodological limitations, coherence, adequacy, and relevance [30]. Where formal grading is not feasible, confidence in the cumulative evidence will be assessed narratively based on study quality, consistency, and contextual relevance.

### Ethics and dissemination

Our review does not require ethical approval because it involves the use of published and publicly available data and does not involve primary data collection. As part of the data extraction process, we will document whether each included primary study reported ethical approval or exemption. The findings of this review will be disseminated through peer-reviewed journal publications, presentations at conferences relevant to primary healthcare and health systems, and policy-oriented outputs. These will include policy briefs and stakeholder engagement activities aimed at supporting implementation decision-making for integrated NCD care within PHC systems in sub-Saharan Africa.

### Patient and public involvement

Patients and members of the public will not be involved in the design of this protocol. However, the findings of the review are intended to inform policymakers, health system managers, and primary healthcare implementers, with the ultimate goal of improving access to and quality of integrated NCD care in sub-Saharan Africa.

## Discussion

SSA is currently navigating a complex health transition characterised by a rising burden of NCDs and persistent infectious diseases [31]. This has increased the pressure on health systems that were historically designed to address acute and episodic conditions. Integrating NCD care into PHC is not only a strategic priority but also a practical necessity for ensuring continuity of care, improving access, and strengthening overall health system performance [32]. Global policy frameworks, including the Declaration of Astana and the WHO PEN, consistently emphasise PHC as the most appropriate platform for delivering integrated, people-centred care [12]. However, translating these policy commitments into effective and scalable implementation strategies remains a significant challenge across the region.

This review is designed to synthesise evidence on how integration of NCD care into PHC is being operationalised in SSA and to identify the strategies that have been employed across diverse contexts. The review adopts a perspective that is particularly relevant to health system strengthening by focusing explicitly on implementation strategies, rather than solely on outcomes. It recognises that the success of integration efforts depends not only on what interventions are introduced, but also on how they are delivered and sustained within specific geographical areas. This approach aligns with the growing emphasis on implementation science in global health, which seeks to bridge the gap between evidence and practice [33].

The anticipated heterogeneity in study designs, intervention types, and outcome measures necessitates the use of a narrative synthesis approach ADDIN CSL_CITATION {“citationItems”:[{”id”:”ITEM-1”,”itemData”:{”DOI”:”10.1097/01.xeb.0000511348.97198.8c”,”ISSN”:”1744–1609”,”abstract”:”Do domestic smoke alarms save lives? Can young offenders be ‘scared straight’ through tough penal measures? What factors should be considered when designing and implementing a multi-sectoral injury prevention programme in a local area? Making sense of large bodies of evidence drawn from research using a range of methods is a challenge. Ensuring that the product of this synthesis process can be trusted is important for policy makers, for practitioners and for the people research is intended to benefit. There are a number of ways in which research evidence can be brought together to give an overall picture of current knowledge that can be used to inform policy and practice decisions. However, the trustworthiness of some of these methods remains problematic. The guidance we set out here focuses on a particular approach – narrative synthesis. Variants of this approach are widely used in work on evidence synthesis, including Cochrane reviews, but there is currently no consensus on the constituent elements of narrative synthesis and the conditions for establishing trustworthiness – notably a systematic and transparent approach to the synthesis process with safeguards in place to avoid bias resulting from the undue emphasis on one study relative to another – are frequently absent. This guidance therefore aims to contribute to improving the quality of narrative approaches to evidence synthesis.”,”author”:[{”dropping-particle”:”“,”family”:”Lisy”,”given”:”Karolina”,”non-dropping-particle”:”“,”parse-names”:false,”suffix”:”“},{”dropping-particle”:”“,”family”:”Porritt”,”given”:”Kylie”,”non-dropping-particle”:”“,”parse-names”:false,”suffix”:”“}],”container-title”:”International Journal of Evidence-Based Healthcare”,”id”:”ITEM-1”,”issue”:”4”,”issued”:{”date-parts”:[[“2016”,”12”]]},”page”:”201”,”publisher”:”Ovid Technologies (Wolters Kluwer Health)”,”title”:”Narrative Synthesis”, ”type”:”article-journal”,”volume”:”14”},”uris”:[“http://www.mendeley.com/documents/?uuid=446d2e3d-a379-3737-b5ae-aebe22cbcbe2”]}],”mendeley”:{”formattedCitation”:” [34]”,”plainTextFormattedCitation”:” [34]”,”previouslyFormattedCitation”:” [33]”},”properties”:{”noteIndex”:0},”schema”:”https://github.com/citation-style-language/schema/raw/master/csl-citation.json”} [34]. This approach allows for the integration of evidence from quantitative, qualitative, and mixed-methods studies, thereby providing a more comprehensive understanding of implementation processes and outcomes. It also enables the identification of patterns across studies, including common barriers such as workforce shortages, limited infrastructure, supply chain constraints, and weak governance, as well as facilitators such as policy support, training and supervision, community engagement, and the use of digital technologies. The review will provide insights into the contextual conditions that influence the success or failure of integration efforts by systematically examining these factors.

The findings of this review are expected to have significant implications for policy and practice by identifying effective implementation strategies and the conditions under which they are most successful. The review will provide actionable evidence to inform the design and scale-up of integrated NCD care within PHC systems in SSA. This is particularly important in the context of efforts to achieve UHC and to meet the targets set under SDG 3, including reducing premature mortality from NCDs and ensuring access to quality essential healthcare services. The review will also highlight gaps in the existing evidence base, thereby informing future research priorities and contributing to the development of more robust and context-sensitive implementation frameworks.

### Limitations

Our protocol has several limitations. The review will be restricted to English-language publications due to resource and feasibility constraints related to the translation and interpretation of non-English full-text articles. This restriction may result in the exclusion of relevant studies from Francophone and Lusophone countries, potentially limiting the geographic representativeness of the findings. Additionally, variability in how integration and implementation strategies are defined and reported across studies may pose challenges for synthesis and comparison. Differences in outcome measurement and reporting may further limit the ability to draw definitive conclusions about effectiveness. The reliance on narrative synthesis, while appropriate for heterogeneous data, may introduce a degree of interpretive subjectivity. These limitations will be addressed through transparent reporting, rigorous methodological procedures, and careful interpretation of findings. Despite these challenges, the review is expected to make a meaningful contribution to the evidence base on health system strengthening in SSA.

## Supporting information

S1 Table PRISMA-P 2015 Checklist. (DOCX)

S1 Text Literature search strategy. (DOCX)

S1 Fig PRISMA flow diagram of study selection process. (DOCX)

S2 Table Quality assessment using the Mixed Methods Appraisal Tool (MMAT). (DOCX)
