# Supplementary material for: Implementation strategies for integrating non-communicable disease care into primary healthcare settings in sub-Saharan Africa: A systematic review protocol
Source: PLoS One. 2026 Aug 14;21(8):e0356003. doi: 10.1371/journal.pone.0356003 (PMC13475893; doi:10.1371/journal.pone.0356003)
Supplement: Supplementary file 2 [file pone.0356003.s002.docx]

**Appendix 1: Draft of search strategy**

**PubMed/MEDLINE**

**Concept blocks:** (NCD terms) AND (PHC terms) AND (integration/implementation terms) AND (sub-Saharan Africa terms)
**Limits:** English; publication date 1990/01/01–2025/12/31.

Draft string (to be revised during piloting):

("Noncommunicable Diseases"[Mesh] OR noncommunicable[tiab] OR "non communicable"[tiab] OR "chronic disease"[tiab] OR "chronic illness"[tiab] OR "mental health"[tiab] OR "mental disorder"[tiab] OR depression[tiab] OR "anxiety disorder"[tiab]

OR hypertension[tiab] OR "high blood pressure"[tiab] OR diabetes[tiab] OR "diabetes mellitus"[tiab] OR "cardiovascular disease"[tiab] OR stroke[tiab] OR cancer[tiab] OR neoplasm[tiab] OR "chronic respiratory disease"[tiab] OR COPD[tiab] OR asthma[tiab])

AND

("Primary Health Care"[Mesh] OR "primary health care"[tiab] OR "primary healthcare"[tiab] OR "primary care"[tiab] OR community health service*[tiab] OR "community health center"[tiab] OR "family practice"[tiab] OR "general practice"[tiab] OR PHC[tiab])

AND

("Delivery of Health Care, Integrated"[Mesh] OR integrat*[tiab] OR "integrated care"[tiab] OR "service integration"[tiab] OR integrated service*[tiab] OR "care integration"[tiab])

AND

(implement*[tiab] OR implementation strateg*[tiab] OR implementation outcome*[tiab] OR "scale up"[tiab] OR "scale-up"[tiab] OR sustain*[tiab] OR "task shifting"[tiab] OR "task sharing"[tiab] OR decentrali*[tiab] OR "guideline implementation"[tiab] OR "quality improvement"[tiab] OR mHealth[tiab] OR "digital health"[tiab] OR telemedicine[tiab])

AND

("Africa South of the Sahara"[Mesh] OR "sub-Saharan Africa"[tiab] OR "sub Saharan Africa"[tiab])

AND

("1990/01/01"[Date - Publication] : "2025/12/31"[Date - Publication])

**Appendix B: Draft Scopus search strategy**

TITLE-ABS-KEY(noncommunicable OR "non communicable" OR "chronic disease*" OR "chronic illness*" OR "mental health" OR "mental disorder*" OR depression OR "anxiety disorder*" OR hypertension OR "high blood pressure" OR diabetes OR "diabetes mellitus" OR "cardiovascular disease*" OR stroke OR cancer OR neoplasm* OR "chronic respiratory disease*" OR COPD OR asthma)

AND

TITLE-ABS-KEY("primary health care" OR "primary healthcare" OR "primary care" OR "community health service*" OR "community health center*" OR "family practice" OR "general practice" OR PHC)

AND

TITLE-ABS-KEY(integrat* OR "integrated care" OR "service integration" OR "integrated service*" OR "care integration")

AND

TITLE-ABS-KEY(implement* OR "implementation strateg*" OR "implementation outcome*" OR "scale up" OR "scale-up" OR sustain* OR "task shifting" OR "task sharing" OR decentrali* OR "guideline implementation" OR "quality improvement" OR mHealth OR "digital health" OR telemedicine)

AND

TITLE-ABS-KEY("sub-Saharan Africa" OR "sub Saharan Africa")

AND

PUBYEAR > 1989

AND

PUBYEAR < 2026

AND

(LIMIT-TO(LANGUAGE, "English"))
