# Supplementary material for: Implementation strategies for integrating non-communicable disease care into primary healthcare settings in sub-Saharan Africa: A systematic review protocol
Source: PLoS One. 2026 Aug 14;21(8):e0356003. doi: 10.1371/journal.pone.0356003 (PMC13475893; doi:10.1371/journal.pone.0356003)
Supplement: Supplementary file 4 [file pone.0356003.s004.docx]

**Mixed Methods Appraisal Tool (MMAT), version 2018**

| **Category of study designs** | **Methodological quality criteria** | **Responses** | | | |
| --- | --- | --- | --- | --- | --- |
|  |  | **Yes** | **No** | **Can’t tell** | **Comments** |
| **Screening questions**  **(for all types)** | S1. Are there clear research questions? |  |  |  |  |
|  | S2. Do the collected data allow to address the research questions? |  |  |  |  |
|  | *Further appraisal may not be feasible or appropriate when the answer is ‘No’ or ‘Can’t tell’ to one or both screening questions.* |  |  |  |  |
| **1. Qualitative** | 1.1. Is the qualitative approach appropriate to answer the research question? |  |  |  |  |
|  | 1.2. Are the qualitative data collection methods adequate to address the research question? |  |  |  |  |
|  | 1.3. Are the findings adequately derived from the data? |  |  |  |  |
|  | 1.4. Is the interpretation of results sufficiently substantiated by data? |  |  |  |  |
|  | 1.5. Is there coherence between qualitative data sources, collection, analysis and interpretation? |  |  |  |  |
| **2. Quantitative randomized controlled trials** | 2.1. Is randomization appropriately performed? |  |  |  |  |
|  | 2.2. Are the groups comparable at baseline? |  |  |  |  |
|  | 2.3. Are there complete outcome data? |  |  |  |  |
|  | 2.4. Are outcome assessors blinded to the intervention provided? |  |  |  |  |
|  | 2.5. Did the participants adhere to the assigned intervention? |  |  |  |  |
| **3. Quantitative non-randomized** | 3.1. Are the participants representative of the target population? |  |  |  |  |
|  | 3.2. Are measurements appropriate regarding both the outcome and intervention (or exposure)? |  |  |  |  |
|  | 3.3. Are there complete outcome data? |  |  |  |  |
|  | 3.4. Are the confounders accounted for in the design and analysis? |  |  |  |  |
|  | 3.5. During the study period, is the intervention administered (or exposure occurred) as intended? |  |  |  |  |
| **4. Quantitative descriptive** | 4.1. Is the sampling strategy relevant to address the research question? |  |  |  |  |
|  | 4.2. Is the sample representative of the target population? |  |  |  |  |
|  | 4.3. Are the measurements appropriate? |  |  |  |  |
|  | 4.4. Is the risk of nonresponse bias low? |  |  |  |  |
|  | 4.5. Is the statistical analysis appropriate to answer the research question? |  |  |  |  |
| **5. Mixed methods** | 5.1. Is there an adequate rationale for using a mixed methods design to address the research question? |  |  |  |  |
|  | 5.2. Are the different components of the study effectively integrated to answer the research question? |  |  |  |  |
|  | 5.3. Are the outputs of the integration of qualitative and quantitative components adequately interpreted? |  |  |  |  |
|  | 5.4. Are divergences and inconsistencies between quantitative and qualitative results adequately addressed? |  |  |  |  |
|  | 5.5. Do the different components of the study adhere to the quality criteria of each tradition of the methods involved? |  |  |  |  |
